# Resection of Cervical Juxtacortical Chondroma and Circumferential Spinal Stabilization for Kyphotic Deformity

**DOI:** 10.7759/cureus.4523

**Published:** 2019-04-22

**Authors:** J. Manuel Sarmiento, Omar Medina, Angelique Sao-Mai S Do, Shimon Farber, Ray M Chu

**Affiliations:** 1 Neurosurgery, Cedars-Sinai Medical Center, Los Angeles, USA; 2 Orthopedic Surgery, Harbor-University of California Los Angeles Medical Center, Los Angeles, USA; 3 Pathology, Cedars-Sinai Medical Center, Los Angeles, USA

**Keywords:** cervical spine, spinal chondroma, cervical kyphosis, circumferential fusion, anterior spine fixation, posterior spine fixation.

## Abstract

Chondromas are rare, benign tumors composed of cartilaginous tissue that mainly affect the metaphases of long tubular bones. Juxtacortical (periosteal) chondromas arise from the surface of periosteum and rarely affect the cervical spine. We present a patient with a spinal juxtacortical chondroma causing spinal cord compression and a cervical deformity treated with surgical resection and circumferential spinal fixation and stabilization.

A 55-year-old female with past medical history of Crohn’s disease with years of neck pain, balance issues, and left upper extremity radicular symptoms. Cervical spine x-rays show kyphosis with an apex at C5, degenerative changes of the endplates and facet joints, and grade 2 anterolisthesis C4 on C5 with no abnormal motion with flexion/extension. MRI showed a left sided C5-6 extramedullary mass measuring 11 x 11 x 15 mm causing spinal cord compression and neural foraminal narrowing. Her pain is worsening and refractory to physical therapy, gabapentin and methocarbamol.

A C4-5 & C5-6 anterior cervical discectomy and fusion, C4-5 & C5-6 laminectomy for tumor resection, and C4-5 & C5-6 posterior fusion with instrumentation was performed. The tumor was completely removed in piecemeal fashion. Microscopic findings showed bland well differentiated cartilaginous neoplasm consistent with juxtacortical chondroma. Postoperative X-rays show partial reduction of C4-5 anterolisthesis and partial reversal of cervical kyphosis. The patient’s radicular pain resolved and neck pain improved postoperatively but she still has some left sided neck pain and hand dysesthesias that are controlled with oral medication one year following surgery.

Cervical chondromas are rare, benign cartilaginous tumors that may present with spinal cord or nerve root compression. They are more complex when they present in patients with co-existing spinal deformities. Maximal safe resection followed by spinal re-alignment and fixation without adjuvant chemotherapy or radiation is recommended in most cases. Close follow-up is recommended to monitor for recurrence.

## Introduction

Chondromas are rare, benign tumors composed of cartilaginous tissue that mainly affect the metaphases of long tubular bones, especially the proximal humerus and distal femur [1-2]. These tumors are categorized as juxtacortical (periosteal) chondromas that arise from the surface of periosteum, enchondromas that arise from within the bone marrow or enchondromatosis, which is a form of osteochondrodysplasia characterized by a proliferation of enchondromas. Juxtacortical chondromas account for <2% of all chondromas and are rarely seen in the cervical spine [3]. We report a case of spinal juxtacortical chondroma causing spinal cord compression with a cervical deformity treated with surgical resection and circumferential spinal fixation and stabilization.

## Case presentation

**Figure 1:**
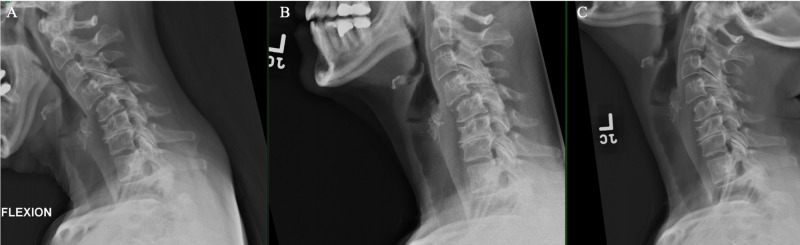
Flexion (A), neutral (B), and extension (C) cervical spine X-rays show kyphosis with an apex at C5, degenerative changes of the endplates and facet joints, and grade 2 anterolisthesis C4 on C5 with no abnormal motion with flexion/extension.

**Figure 2:**
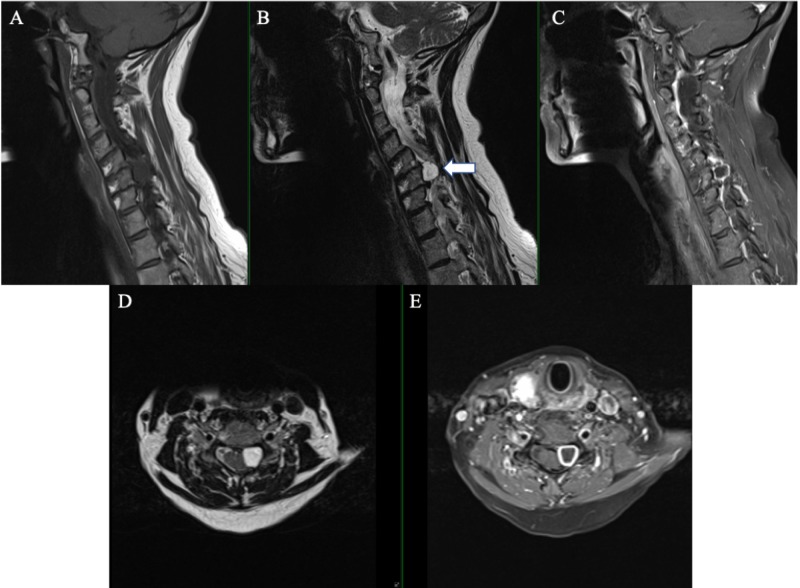
Magnetic resonance imaging showing a left sided well-circumscribed 11 x 11 x 15 mm extramedullary tumor (arrow) that was hypointense on sagittal T1-weighted image (A), hyperintense on sagittal T2-weighted image (B), enhancing peripherally with gadolinium in the sagittal plane (C). On the axial plane the tumor showed hyperintense on T2-weighted image (D) and peripheral enhancement with gadolinium (E).

A 55-year-old female with past medical history of Crohn’s disease controlled with IV infusions of golimumab, a TNF alpha inhibitor, presents with years of worsening neck pain and left arm pain with associated numbness in a C6 distribution that is refractory to physical therapy and conservative therapy. She has been taking prednisone intermittently for years due to her Crohn’s disease. She complains of dropping objects with the left hand and having recent difficulties with balance. There is no associated lower extremity numbness nor bowel/bladder incontinence. She is taking methocarbamol on an as-needed basis for muscle spasms and gabapentin for neuropathic pain. On physical examination, she is full strength on all extremities with negative Hoffman’s sign and normal plantar flexion reflex. Spurling’s sign is absent and Lhermitte’s sign is negative. Cervical spine X-rays show kyphosis with an apex at C5, degenerative changes of the endplates and facet joints, and grade 2 anterolisthesis C4 on C5 with no abnormal motion with flexion/extension (Figure 1). Magnetic resonance imaging (MRI) cervical spine showed a left-sided C5-6 extramedullary mass measuring 11 x 11 x 15 mm causing spinal cord compression and neural foraminal narrowing (Figure 2). The mass exhibits hypointensity on T1-weighted images, hyperintensity on T2-weighted images, and homogenous peripheral enhancement.

Surgical approach

We planned for a C4-5 & C5-6 anterior cervical discectomy and fusion, C4-5 & C5-6 laminectomy for tumor resection, and C4-5 & C5-6 posterior fusion with instrumentation. The patient was first positioned supine on the operating room table and a right transverse cervical incision hidden in a skin fold in the neck centered at the cricoid cartilage was made. The C5-6 level was reached with a standard anterior cervical approach and confirmed with fluoroscopy. Caspar pins were placed for vertebral body distraction followed by discectomy and placement of an 8-mm Synthes Zero P standalone spacer (DePuy Synthes, Pennsylvania, USA) loaded with demineralized bone matrix allograft and autograft from drilling. The same steps were performed for the C4-5 interspace. The patient was then positioned prone in Mayfield pins for the posterior approach. Pilot holes for lateral mass screws were drilled prior to laminectomy so that the decompression does not interfere with posterior cervical landmarks. A complete laminectomy was made with a high-power drill (Medtronic, Minnesota, USA) at C5/C6 and a partial laminectomy was made at C4. There was a gray, fibrous and moderately vascular epidural mass arising from the left lateral spinal canal that was resected in piecemeal fashion. The mass was swept from the ventral side of the spinal dura and ventral to the C5 and C6 nerve roots. The trajectory for lateral mass screws was aimed 20 degrees lateral from perpendicular to the lateral mass and 30 degrees cephalad paralleling the facet joints. Six 14-mm screws (DePuy Synthes, Pennsylvania, USA) were placed at bilateral lateral masses from C4 to C6 followed by placement of custom rods that were locked and tightened. The lateral masses and facets were decorticated; autologous bone graft from the spinous processes and lamina was morselized and placed over the decorticated surfaces. Neurophysiologic monitoring of somatosensory-evoked potentials remained at baseline throughout the operation.

Pathology

**Figure 3:**
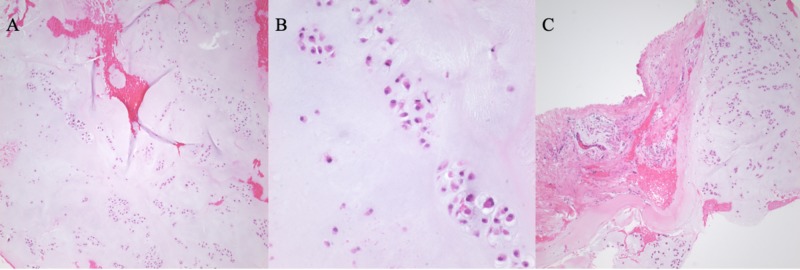
Hematoxylin and eosin stain in low magnification shows lobulated areas of the bland, cartilaginous neoplasm (A); high magnification shows minimal cytologic atypia and two binucleated chondrocytes (B); low magnification shows smooth interface between periosteum and cartilaginous tissue (C).

**Figure 4:**
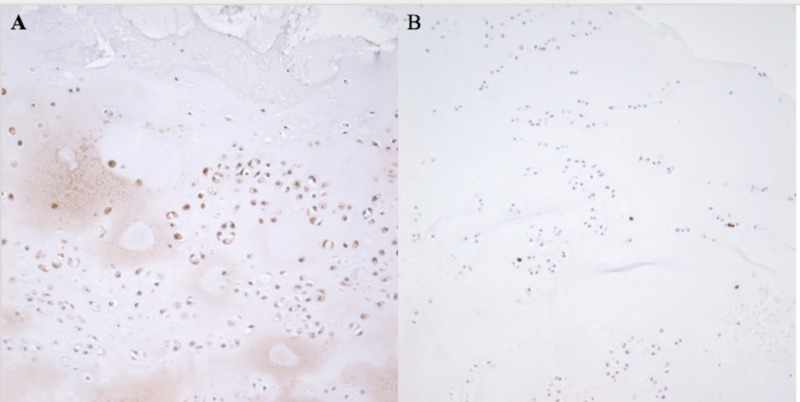
Immunohistochemistry showing S100 positivity in chondroid tumor cells (A); immunohistochemistry showing rare cells with positive Ki-67 nuclear expression (B).

The specimen was submitted in two parts, with multiple fragments of gray, smooth-surfaced, glistening semi-transparent soft tissue aggregating to 1.5 x 1.0 x 1.0 cm. Microscopic examination revealed lobules of hyaline cartilage surrounded by a periosteum layer (Figure 3). There was focal hypercellularity and myxoid change, and high power revealed minimal cytologic atypia with minimal nuclear abnormalities, and few binucleated cells. Immunohistochemistry was positive for S100, and 3% of cells were positive for Ki-67 nuclear staining (Figure 4). The findings were correlated with a musculoskeletal radiologist to confirm a diagnosis consistent with juxtacortical chondroma.

Postoperative course

**Figure 5:**
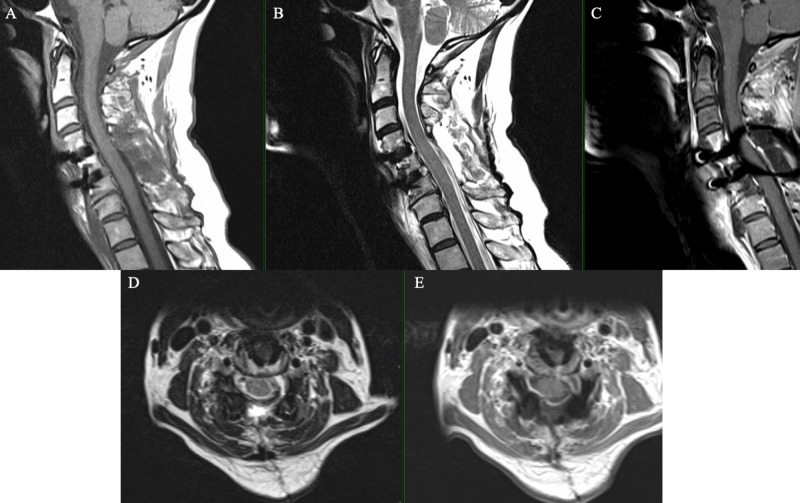
Magnetic resonance imaging showing a gross-total resection of tumor on sagittal T1-weighted image (A), hyperintense on sagittal T2-weighted image (B), sagittal T1-weighted image with gadolinium in the sagittal plane (C). On the axial plane, there is gross-total resection of tumor on T2-weighted image (D) and T1-weighted image with gadolinium (E).

**Figure 6:**
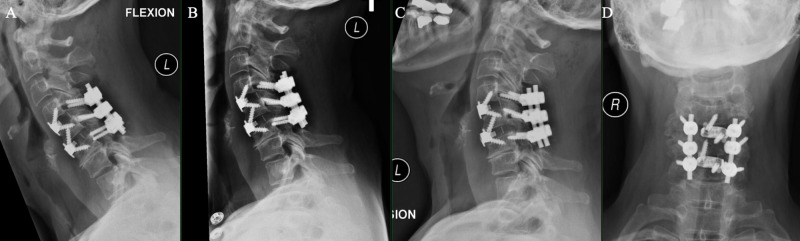
Flexion (A), neutral (B), extension, (C) and coronal (D) cervical spine X-rays showed screws and hardware in proper position with partial reduction of C4-5 anterolisthesis and partial reversal of cervical curvature.

**Figure 7:**
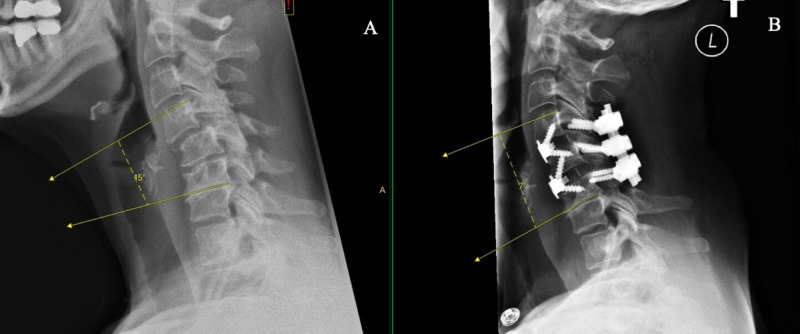
Lateral cervical spine X-rays showing preoperative sagittal Cobb angle of +15 (A) and postoperative Cobb angle of +7 (B).

Postoperative MRI showed gross total resection of the tumor (Figure 5) and cervical x-rays showed screws and hardware in proper position with partial reduction of C4-5 anterolisthesis and partial reversal of cervical curvature (Figure 6). The sagittal Cobb angle improved from +15 preoperatively to +7 postoperatively (Figure 7). The C4 cephalad endplate and C6 caudal endplate were used to calculate Cobb angles. She remains neurologically intact with significantly improved left sided sharp radicular pain but has persistent left hand dysesthesia and left sided neck pain requiring relief with gabapentin and occasionally methocarbamol one year after surgery. Her balance and ambulation have improved. Follow-up MRI at one year showed no tumor recurrence.

## Discussion

Etiology

Chondromas are uncommonly seen in the cervical spine. A recent review of the literature indicates there have been 18 reported cases of cervical chondroma since 1960 [4]. In this review, Inoue et al. highlight that the cervical chondromas may be derived from the vertebral body, lateral mass, lamina, pedicle, transverse process or spinous process [4]. Furthermore, while all cervical spinal levels may be affected, there is an apparent proclivity towards originating from the subaxial spine levels C4, C5, and C6. Spinal chondromas are thought to be derived from hyperplasia of immature spinal cartilage that has migrated outside the vertebral axis or from metaplasia of the cartilaginous tissue in contact with the spinal elements or annulus fibrosus [5]. Spinal chondromas usually remain asymptomatic so long as they are confined within the vertebral elements. Neurologic signs and symptoms may develop with tumor growth causing increasing mass effect and compression of the spinal cord and nerve roots.

Radiology

Although not evident in our patient, X-rays may show a well-circumscribed lytic lesion and possibly a widened neural foramen for intraforaminal tumors [6-7]. Due to the rarity of this tumor in the spine the authors do not feel that a modern screening protocol for chondromas is practical and does not warrant the use of limited resources and the risk of exposure to extra radiation. However, asymptomatic patients with a known osseous spinal tumor should have annual follow-up for monitoring of tumor size and morphology. Computed tomography (CT) can be useful for characterizing the osseous structures surrounding the tumors and may show a radiolucent, erosive lesion [8]. MRI is useful to ascertain the relationship between the tumor, spinal cord, exiting nerve roots, and nearby ligaments. Chondromas are typically well-circumscribed lesions with intermediate signal intensity and heterogenous peripheral enhancement with gadolinium on T1-weighted images [9]. High signal intensity on T2-weighted image is observed due to the high water content of hyaline cartilage that composes the tumor [7].

Pathology, treatment, and recurrence

Spinal chondromas are comprised of neoplastic chondrocytes within a backdrop of hyaline or myxoid cartilage [8]. Immunohistochemistry stains were negative for SOX10 and Pancytokeratin which ruled out a chordoma. However, our patient’s tumor was positive for S100 as expected for a chondroma. Ki-67 expression was extremely low indicating a high likelihood of a benign process. Surgery is indicated for cervical spinal chondromas when neurological disabilities or deficits arise that impair normal function. Complete surgical excision of the tumor is necessary, as tumors may recur if cartilage is left behind. Even in cases of complete resection, recurrence has been observed in 14% of cervical spinal chondromas [4]. Malignant transformation of vertebral chondromas may even occur, as reported by Morard et al [10]. Therefore, close follow-up is required in the postoperative period for all patients with cervical chondromas. Adjuvant chemotherapy is ineffective for cervical chondromas, and radiation therapy should be considered in patients with unresectable tumors or in cases in which surgical boundaries are histologically positive for tumor cells [11].

Spinal fusion

 We present a patient with a C5 vertebral chondroma causing spinal cord compression and radicular symptoms in a patient with kyphotic deformity and multi-level degenerative disc disease likely secondary to chronic glucocorticoid steroid treatment. Surgical fusion with instrumentation following tumor resection was necessary in this case to restore cervical lordosis, prevent further kyphosis, re-align, and stabilize the spine. An alternative treatment is a left-sided hemilaminectomy without instrumentation and fusion. However, this approach poses a more challenging (and narrow) surgical corridor for tumor resection and does not address this patient’s apparent cervical deformity. Instead, a two-level anterior and posterior, or circumferential, fusion was preferred in this case to achieve optimal deformity correction, prevent spinal destabilization at the C4-5 anterolisthesis following a C5 laminectomy, and ensure a rigid construct for maximal spinal immobilization without restricting the patient to further loss of spinal motion with a longer construct. An anterior cervical approach was performed first to place interbody grafts and improve cervical lordosis before locking the construct in place from a posterior approach with lateral mass screw fixation. Locking of the rod-screw system also generates a cantilever force that improves cervical lordosis. The lower level was approached first in this case because it is generally easier to access higher interbody levels than lower levels after interbody graft placement. Over a quarter (5/18, 27.8%) of reported cervical chondroma cases required some form of spinal fixation [4-5,12-14]. It is important to remember that spinal fixation may still be needed in the absence for cervical deformity if the spine becomes de-stabilized in the process of tumor resection (e.g., if more than half of a facet joint is removed).

## Conclusions

Cervical chondromas are rare, benign cartilaginous tumors that may present with spinal cord or nerve root compression in patients with kyphotic deformities. Maximal safe resection followed by spinal re-alignment and fixation without adjuvant chemotherapy or radiation is recommended in most cases. Close follow-up is recommended to monitor for recurrence.
